# Limb girdle muscular dystrophy 23 caused by compound heterozygous mutations of *LAMA2* gene

**DOI:** 10.3389/fped.2023.1191068

**Published:** 2023-06-19

**Authors:** Yuqing Xu, Linyan Zhu, Yeqing Qian, Minyue Dong

**Affiliations:** ^1^ Women’s Hospital, School of Medicine, Zhejiang University, Hangzhou, China; ^2^ Key Laboratory of Reproductive Genetics, Ministry of Education (Zhejiang University), Hangzhou, China; ^3^ Department of Obstetrics and Gynaecology, Ningbo First Hospital, Ningbo, China

**Keywords:** *LAMA2*, *LAMA2*-related CMD, limb girdle muscular dystrophy 23, whole exome sequencing, LamG domain

## Abstract

**Introduction:**

Mutations of *LAMA2* gene are associated with congenital muscular dystrophy (CMD). The *LAMA2*-related CMD mainly consists of two diseases, merosin deficient congenital muscular dystrophies type 1A (MDC1A) and limb girdle muscular dystrophy 23 (LGMD23). LGMD23 is characterized by slowly progressive proximal muscle weakness, which primarily affects the lower limbs and results in gait difficulties. Additional clinical features include increased serum creatine kinase, abnormal electromyography with or without white matter abnormalities on brain imaging.

**Methods:**

Clinical data were collected from a Chinese Han family. Whole-exome sequencing, Sanger sequencing, RT-PCR and TA clone sequencing were performed on the family members.

**Results:**

Compound heterozygous mutations of *LAMA2*: c.1693C > T (*p*. Q565*) (maternally inherited) and c.9212-6T > G (paternally inherited) were identified and confirmed in the proband. The mutation c.1693C > T (*p*. Q565*) was classified as pathogenic according to American College of Medical Genetics and Genomics (ACMG) guidelines. By performing RT-PCR and TA clone sequencing, an insertion of 40-bp intronic sequence (intron 64) was found in the transcripts of the proband and her father, which resulted in a frameshift and premature truncation codon of the *LAMA2*. In particular, the variant truncated the LamG domain of the LAMA2. Therefore, the c.9212-6T>G was classified as likely pathogenic according to American College of Medical Genetics and Genomics (ACMG) guidelines.

**Discussion:**

Our findings described two novel mutations in a girl with LGMDR23, which contributes to the genetic counseling of the family and expands the clinical and molecular spectrums of the rare disease.

## 1. Introduction

Laminin is a large family of heterotrimeric glycoproteins which contribute to cell adhesion, differentiation, neurite outgrowth and extracellular matrix architecture (1). Laminin subunit α2(LAMA2), belonging to the laminin family, interacts with integrin α7β1 (2, 3) and dystroglycan with the large globular(G) C-terminal domain. LAMA2 protein connects other laminins with its N-terminal domain to form a protein meshwork, which is helpful for the supramolecular assembly of the basal membrane (4, 5). The mutations in the *LAMA2* gene caused congenital muscular dystrophy (CMD), an autosomal recessive disorder. The prevalence of CMDs are estimated to be 0.563 per 100,000 in Italy (6) and 0.017–0.083 per 100,000 in China (7). LAMA2-realted CMD ranges from severe, early onset merosin deficient congenital muscular dystrophies type 1A (MDC1A, OMIM 607855), to mild, limb-girdle muscular dystrophies (LGMDR23, OMIM 618138) (8, 9).

**Table 1:** Clinical and laboratory data of the proband.

Items	Patient
Gender	Female
Age	9-year-old
Age of first admission	3-year-old
Creatine kinase (U/L) (Normal range: 24–229)	1,583
Head and Neck	Chewing difficulty
Skeletal system	The prominent scapula
Musculature	Myogenic damage Motor delay No pseudohypertrophy of tongue and calf muscles Independent walking at 3–6 years of age Toe walking from 6 years of age
Growth development	Delay Normal verbal cognition
Nervous system	No history of seizure
Respiratory system	Transient pneumonia at 6 years old
Digestive system	Normal

Patients with MDC1A often suffer from hypotonia and severe proximal weakness at birth, as well as early respiratory and feeding difficulties. Furthermore, joint contractures, kyphoscoliosis and seizure are usually recognized. Compared with MDC1A, LGMDR23 is characterized by a milder and later age of onset, ranging from late childhood to mid-adulthood (10–12). Increased serum creatine kinase level, abnormal electromyography and abnormal white matter signal on brain magnetic resonance imaging (MRI) can be identified in both diseases.

Herein, we described a Chinese girl with LGMDR23 caused by two novel mutations in the *LAMA2* gene.

## 2. Methods

### 2.1. Subject

A healthy non-consanguineous couple and their 9-year-old girl were referred to the Department of Reproductive Genetics, Women's Hospital, School of Medicine Zhejiang University in October, 2022. The girl (the proband) was born at term via virginal with an Apgar score of 10. Her development history was unremarkable until she was 3 years old. She was then firstly noted for being clumsy in general, as well as slow and awkward in her running. She had continued difficulties in jumping, running, lifting heavy objects and climbing stairs over time. Her serum creatine kinase was 1583 U/L (reference range: 24–229 U/L). Electromyography showed myogenic damage in limbs. Her language and learning development were normal. No family history of motor difficulties was observed. At the age of 6 years, she suffered from pneumoniae and recovered. Since then, she has achieved independent ambulation. In addition, she presents with toe walking and has difficulty in chewing due to masseter muscle weakness (Table 1).

This study was approved by the Institutional Review Board of the Women's Hospital, School of Medicine, Zhejiang University.

### 2.2. Whole-exome sequencing

The whole-exome sequencing(WES) was performed as previously described (13).

### 2.3. Sanger sequencing

The Sanger sequencing was conducted as previously described (13). The primers for c.1693C > T were 5′- ACAATGGAAGCCTATGTGAG -3′ and 5′- TGTGATTTAGCTGGTTCTGG -3′. The primers for c.9212-6T > G were 5′- ACACTTTGGGCATAGATGGG -3′ and 5′- CTTTGGTGAGCTTCAGGGAT -3′.

### 2.4. RNA analysis and TA clone sequencing

The RT-PCR experiment was conducted as previously described (13). The PCR primers were 5′-ACAACGACTGGAGTTCTTCT -3′ and 5′-TCCTGGGGTTACACTTATTT -3′. The PCR products were separated by electrophoresis on 2.0% agarose gel. Little difference was observed between the two mRNA splicing products, which caused difficulties for sequencing. TA cloning was chosen to purify the PCR products with the HieffClone^TM^ Zero TOPO-TA Cloning Kit (Yeasen, China). And then the purified PCR product was selected for sequencing.

## 3. Results

### 3.1. Compound heterozygous variants of *LAMA2* gene

**Figure 1:**
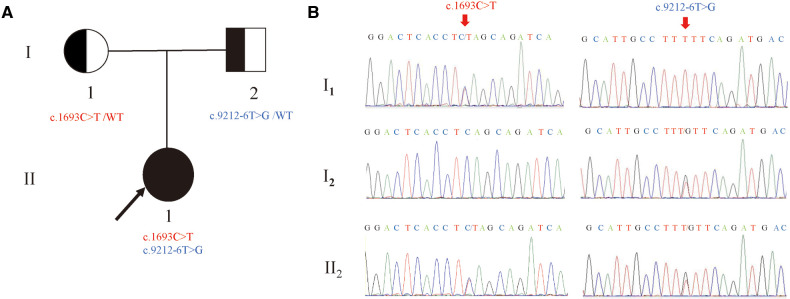
Newly identified nonsense and splicing variants in the *LAMA2* gene. (**A**) The pedigree of the family.II_1_ (the proband) have two compound variants (c.1693C > T and c.9212-6T > G) in *LAMA2* gene, which are inherited from the I_1_(mother) and the I_2_(father), respectively. (**B**) Sanger sequencing analysis. The two variants(c.1693C > T and c.9212-6T > G) were validated by Sanger sequencing. (red arrows indicated the mutation).

The compound heterozygous variants of *LAMA2*: c.1693C > T (p.Q565*) and c.9212-6T > G were identified in the proband by whole-exome sequencing (Figure 1). Sanger sequencing confirmed that the c.1693C > T (p.Q565*) and c.9212-6T > G were inherited from the mother and the father, respectively. Therefore, the parents were both carriers and the co-segregation of genotype and phenotype was in accordance with autosomal recessive inheritance (PP4).

Neither of the variants had been reported in database (gnomAD, ClinVar or HGMD) or literature (PM2). The *LAMA2*: c.1693C > T (p.Q565*) variant theoretically introduces a stop codon and a truncated protein (PVS1). According to ACMG recommendations, the mutation *LAMA2*: c.1693C > T (p.Q565*) was classified as pathogenic (PP4 + PM2 + PVS1), while the variant *LAMA2*: c.9212-6T > G was classified as variant of uncertain significance (VUS).

### 3.2. Pathogenicity of *LAMA2*: c.9212-6T > G

**Figure 2:**
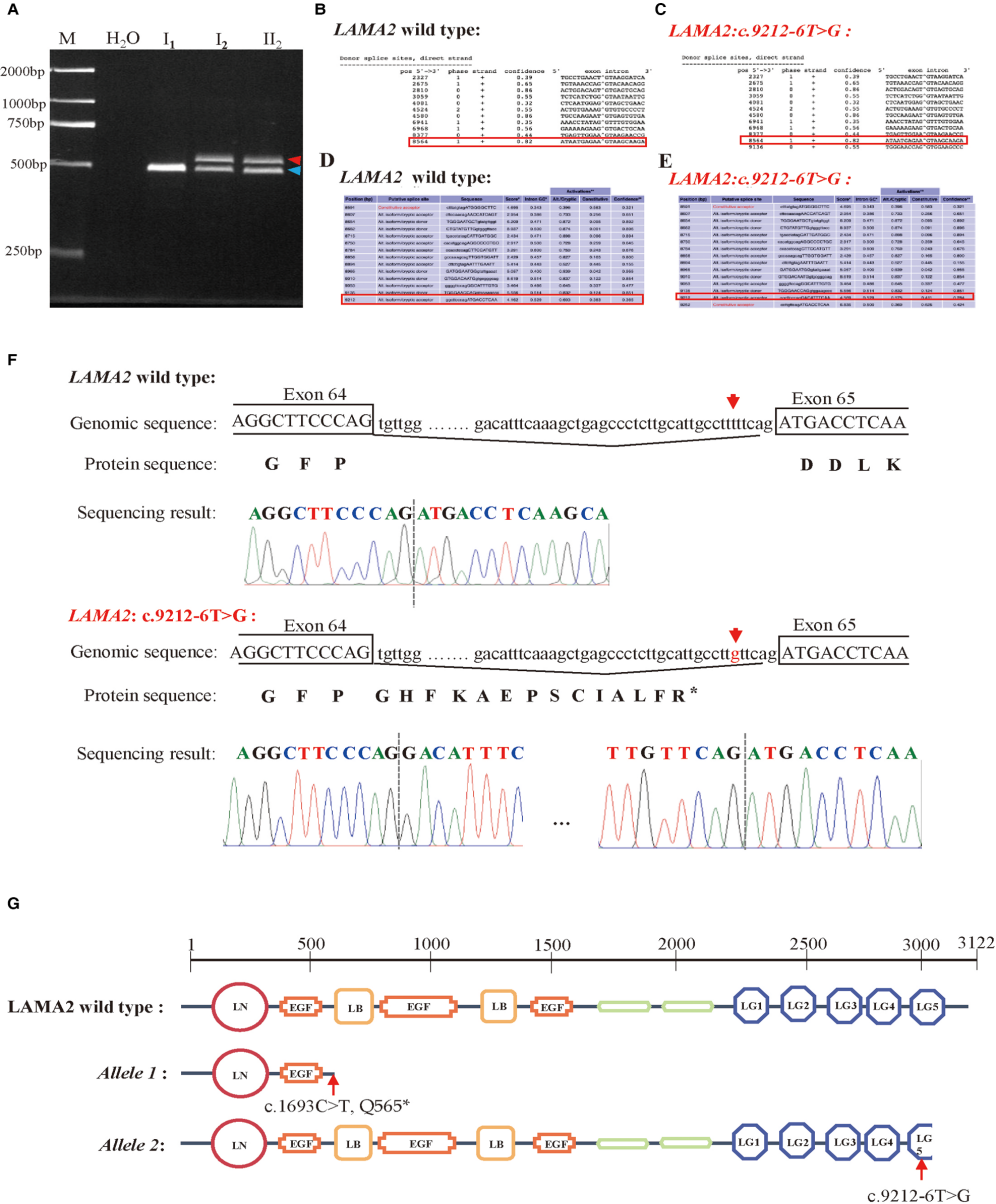
Analysis of the c.9212-6T > G variant in the *LAMA2* gene. (**A**) RT-PCR analysis of *LAMA2* cDNA from peripheral blood samples of the family members. Agarose gel (2.0%) electrophoresis of RT-PCR products generated from I_1_(mother), I_2_(father) and II_1_(the proband). Aberrantly spliced mRNA and normal spliced mRNA are marked by red and blue arrowheads, respectively. (**B–E**) Predictive results of the c.9212-6T > G variant site in splicing. (**B**) The Predictive result of wild type *LAMA2* by using NetGene2 Server (the red square represented). (**C**) The Predictive result of *LAMA2*: c.9212-6T > G by using NetGene2 Server (the red square represented). (**D**) The Predictive result of wild type *LAMA2* by using ASSP tool (the red square represented). (**E**) The Predictive result of *LAMA2*: c.9212-6T > G by using ASSP tool (the red square represented). (**F**) TA clone sequencing and sequencing analysis of the RT-PCR products from the proband. The red arrows reveal the position of the c.9212-6T > G variant. (**G**) Diagrams of domains of wild-type, c.1693C > T (p.Q565*) variant and c.9212-6T > G variant of *LAMA2*. The *LAMA2:* c.1693C > T (p. Q565*) has only two domains and the LG5 domain of *LAMA2: c*.9212-6T > G is truncated compared with wild type *LAMA2*.

NetGene2 Server (http://www.cbs.dtu.dk/services/ NetGene2/) and Alternative Splice Site Predictor (ASSP) *(*http://wangcomputing.com/assp/index.html*)* were used to predict the effect of the variant c.9212-6T > G on splicing. It was predicted that the variant affected splicing (Figure 2B–E). For the validation, RNAs were extracted from peripheral blood of the proband and her parents. cDNAs were then reverse transcribed to amplify exons 63–65 of *LAMA2* with the primers. 2.0% agarose gel electrophoresis demonstrated that the proband and her father had a larger transcript than the normal amplification fragment (Figure 2A). Further results of TA clone sequencing and sequencing showed that the larger transcript had 40–bp intron 64 before the exon 65, which caused a truncation of LAMA2 by a frameshift and creation of a premature termination codon (Figure 2F). As was showed in Figure 2G, the splicing mutation (c.9212-6T > G) was predicted to generate prematurely truncated LAMA2 in the last LamG domain. The truncated effect does cause LAMA2 deficiency and damage the ability to interact with integrin α7β1 and dystroglycan. Considering the accordance between phenotype and genotype and the rarity of the splicing variant, the mutation *LAMA2:* c.9212-6T > G was classified as likely pathogenic (PP4 + PM2 + PS3) according to ACMG guidelines.

## 4. Discussion

In the current investigation, we reported a Chinese girl diagnosed as LGMDR23 due to the compound heterozygous variants in the *LAMA2* gene. The maternally inherited mutation c.1693C > T (p.Q565*) of *LAMA2* was pathogenic and the paternally inherited mutation c.9212-6T > G was proved to be a splicing mutation which resulted in frameshift and truncation. To the best of our knowledge, both of the variants have never been reported in any database or literature, indicating our findings extend the mutation spectrum and clinical knowledge of the diagnosis for the LGMDR23.

The patients with CMD due to laminin anomaly presented with severe muscle weakness, hypotonia and white matter abnormalities in brain imaging (14–16). In addition, all the patients could not achieve independent ambulation. More variabilities in the phenotype associated with *LAMA2* deficiency were described thereafter. Compared with the severe features, some patients were characterized by milder and later-onset muscle weakness, slightly elevated creatine kinase level, ability to achieve ambulation, with or without abnormal brain imaging (17, 18). By carrying out muscle biopsies and immunocytochemistry with antibodies against 80 and 300 kDa fragment of LAMA2, complete absence of LAMA2 expression was found in patients with severe phenotype while reduced LAMA2 expression was found in those patients with mild phenotype. Therefore, LAMA2-related muscular dystrophy (MD) is generally divided into MDC1A (also named LAMA2-CMD, the severe one) and LGMDR23(the mild one) categories.

LGMDR23 is much rarer than MDC1A in China. Even in the largest and multicenter research of LAMA2-related muscular dystrophy in 2021, 14 cases of LGMDR23 were reported while 116 cases of MDC1A were reported (19). The symptoms of LGMDR23 included myopathic gait, difficulties in running and jumping, and epilepsy. Moreover, the median age of onset for LGMDR23 was 18.0 months (range 13.0–156.0 months) (19). In the current investigation, the proband was firstly noted for being clumsy and being slow and awkward in her running at 3 years old, belonging to the late-onset of LAMA2-related MD. She also had other typical symptoms like elevated creatine kinase level, transient pneumonia, delayed motor milestones, difficulty in chewing and abnormal electromyography. Additionally, the proband presented with toe walking which was also observed in a Turkish origin male at 10 years old (20) and in a Chinese origin female at 4 years old and a male at 6 years old (21).

The variants c.1693C > T (p.Q565*) and c.9212-6T > G of *LAMA2* gene were detected by WES and confirmed by Sanger sequencing. These two variants were inherited from her mother and father, respectively. Based on ACMG guidelines, the nonsense c.1693C > T (p.Q565*) variant was classified as pathogenic while the c.9212-6T > G was classified as VUS. To date, approximately 100 nonsense variants and 90 splicing variants of *LAMA2* have been described, without obvious mutational hotspots (22). After analysis of the genotype-phenotype correlations of the MDC1A and LGMDR23, Dandan et al. concluded that nonsense, frameshift or copy numbers variant caused more damage than splicing or missense variants (19).

With the highly genotype-phenotype correlation and predicted results of NetGene2 Server and ASSP, RT-PCR, TA clone sequencing and sequencing were performed to investigate the pathology of the splicing mutation. The c.9212-6T > G variant led to 40-bp intron insertion before the exon 65, which caused frameshift and truncation of the *LAMA2* (Figure 2F). Thus, the significant LamG domain at the C-terminus of LAMA2 was truncated, which might damage the linkage between the extracellular matrix and the dystrophin-glycoprotein. The results upgraded the pathogenicity evidence of the c. c.9212-6T > G variant, so the variant classification was changed from VUS to likely pathogenic. In the meanwhile, because the splicing variant still allow partial expression of LAMA2 gene, it might explain the mild and late on-set phenotype in the proband like those patients in a previous published study (23). However, muscle biopsy was not performed for further study because we could not get the permission from the family to obtain the sample.

In summary, we described a Chinese girl diagnosed as LGMDR23 due to typical clinical phenotypes and laboratory tests, and identified two novel variants of *LAMA2* gene via WES and Sanger sequencing. By performing RT-PCR,TA clone sequencing, we demonstrated the variant c.9212-6T>G was a likely pathogenic splicing mutation. Our findings expand the mutation spectrum and provided information for the genetic counseling of LGMDR23.

## Data Availability

The original contributions presented in the study are publicly available. This data can be found here: https://ngdc.cncb.ac.cn/bioproject/browse/PRJCA013814.
